# Heavily Gd-Doped Non-Toxic Cerium Oxide Nanoparticles for MRI Labelling of Stem Cells

**DOI:** 10.3390/molecules28031165

**Published:** 2023-01-24

**Authors:** Anton L. Popov, Irina V. Savintseva, Taisiya O. Kozlova, Olga S. Ivanova, Ivan V. Zhukov, Alexander E. Baranchikov, Alexandra V. Yurkovskaya, Andrey A. Savelov, Artem M. Ermakov, Nelli R. Popova, Konstantin L. Ivanov, Vladimir K. Ivanov

**Affiliations:** 1 Institute of Theoretical and Experimental Biophysics of the Russian Academy of Sciences, 3 Institutskaya St., Pushchino 142290, Russia; 2 Kurnakov Institute of General and Inorganic Chemistry of the Russian Academy of Sciences, 31 Leninskiy Prosp., Moscow 119991, Russia; 3 International Tomography Center, Siberian Branch of the Russian Academy of Sciences, 3A Institutskaya St., Novosibirsk 630090, Russia

**Keywords:** apoptosis, ceria, gadolinium, MRI contrast agents, nuclear magnetic resonance, proliferative activity, solubility, stem cells

## Abstract

Recently, human mesenchymal stem cells (hMSc) have attracted a great deal of attention as potential therapeutic agents in the treatment of socially significant diseases. Despite substantial advances in stem-cell therapy, the biological mechanisms of hMSc action after transplantation remain unclear. The use of magnetic resonance imaging (MRI) as a non-invasive method for tracking stem cells in the body is very important for analysing their distribution in tissues and organs, as well as for ensuring control of their lifetime after injection. Herein, detailed experimental data are reported on the biocompatibility towards hMSc of heavily gadolinium-doped cerium oxide nanoparticles (Ce_0.8_Gd_0.2_O_2−x_) synthesised using two synthetic protocols. The relaxivity of the nanoparticles was measured in a magnetic field range from 1 mT to 16.4 T. The relaxivity values (*r*_1_ = 11 ± 1.2 mM^−1^ s^−1^ and *r*_1_ = 7 ± 1.2 mM^−1^ s^−1^ in magnetic fields typical of 1.5 and 3 T MRI scanners, respectively) are considerably higher than those of the commercial Omniscan MRI contrast agent. The low toxicity of gadolinium-doped ceria nanoparticles to hMSc enables their use as an effective theranostic tool with improved MRI-contrasting properties.

## 1. Introduction

Magnetic resonance imaging (MRI) provides remarkable possibilities for the visualisation of soft tissues and physiological processes in the human body. MRI scanners are widely available in hospitals, enabling rapid diagnosis of various dysfunctions, including those of the brain, abdomen and spinal cord. To increase the contrast in MRI images, various contrast agents are commonly used, which change the longitudinal (*T*_1_) and transverse (*T*_2_) relaxation times of water protons. Gd-based compounds are the most effective magnetic resonance imaging contrast agents [1,2] and now almost 50 tons of gadolinium are administered annually [3]. The high efficiency of these compounds is due to the fact that Gd^3+^ has an extremely high magnetic moment (7.94 μB), since seven unpaired electrons (symmetric ^8^S_7/2_ ground electronic state) provide sufficiently high (10^−8^–10^−9^ s) electronic relaxation times. Currently, FDA- and EMA-approved (FDA, U.S. Food and Drug Administration; EMA, European Medicines Agency) Gd-based contrast agents include gadobenate (MultiHance), gadobutrol (Gadavist), gadodiamide (Omniscan), gadopentetate (Magnevist), gadoterate (Dotarem), gadoteridol (ProHance) and some others [4]. One serious problem associated with the use of Gd-containing MRI contrast agents is the lability of gadolinium complexes and the interaction of free Gd^3+^ ions with body tissues (*inter alia*, with brain tissues), which can cause systemic nephrogenic fibrosis (NFS) after repeated use [4]. Furthermore, the safety of some approved contrast agents (e.g., Gadavist or Magnevist) is questionable, and is being re-considered [1,4]; a number of countries, including Japan and Great Britain, have refused to use linear Gd-containing MRI contrast agents [5]. Thus, there is an acute need for a new generation of MRI contrasts that are safe, have high selectivity in tissue accumulation and provide a strong signal in MRI imaging, for diagnostic magnetic resonance measurements.

In the authors’ previous studies, a proposal was made for the design of new Gd-based MRI contrast agents based on nanocrystalline Gd-doped ceria [6,7,8,9]. In an oxide matrix, Gd^3+^ ions can provide higher values of longitudinal relaxation constants than Gd^3+^ in chelate complexes. Furthermore, poorly soluble gadolinium oxide compounds have been reported to have low toxicity [10,11]. Ceria matrix not only has an extremely low solubility over a wide pH range [12], but is also fully compatible with cells and tissues, protecting them from negative external factors, e.g., oxidative stress, the action of ionising radiation, etc. [13,14]. In recent reports, cerium dioxide nanomaterials have been identified as being promising materials for cancer theranostics [8,15,16,17]. Previously, the possibility of synthesising cerium dioxide nanoparticles (NPs) heavily doped with gadolinium was shown, as was their possible use as an MRI contrast agent [6,8,9,18]. Eriksson et al. [19] also showed that Gd-doped CeO_2_ NPs can act as an antioxidant agent, while having *r*_1_ relaxivities from 7 to 13 mM^–1^·s^–1^.

In cancer treatment, the use of human mesenchymal stem cells (hMSc) as delivery systems for therapeutic agents has been shown to open up new therapeutic horizons [20], since it bypasses known problems in the targeted delivery of biologically active substances, using various nanovehicles, (to overcome, for example, the binding site barrier [21]). It was previously shown that hMSc expressing TRAIL (tumour necrosis factor related apoptosis inducing ligand) can provide targeted delivery of this proapoptotic agent directly to breast cancer metastases [22]. Nakamizo et al. showed that hMSc enter human gliomas after intravascular or local delivery and significantly increase the survival rate of animals with human U87 intracranial glioma xenografts [23]. Bone-marrow hMSc delivered to the tumour site and forcibly overexpressing IFN-β inhibited the growth of malignant cells in vivo. Importantly, this effect required the integration of hMSc directly into the tumour, and could not be achieved with systemically delivered IFN-β or IFN-β produced by hMSc at a site far from the tumour [24]. Thus, the ability of hMSc to invade tumour tissues and specifically locate in them, affecting tumour cells, suggests their use as a delivery vehicle for various anticancer agents, and in vivo hMSc imaging methods will be required to control the efficacy of such therapy. Such an approach was used for MRI imaging of hMSc loaded with superparamagnetic iron oxide nanoparticles to track multiple lung metastases in vivo [25].

On the other hand, hMSc-based therapies were reported to be limited, due to the possible malignant transformations of hMSc [26]. These transformations can also be monitored using advanced MRI techniques, e.g., using tumour specific promoters [26]. Another important task, the visualisation of cancer stem cells, being a rare but highly resistant subpopulation of malignant cells, is also an emerging topic in cancer treatment, since these cells possess similar characteristics to normal stem cells, as well as the substantial ability to self-renew and for extensive proliferation [27,28].

In view of these points, the search for effective MRI contrast agents capable of being administered into the human body for stem cell visualisation is of great importance for various biomedical applications. These applications include high resolution imaging of stem cells’ localisation in tissues, cancer therapies and in vivo understanding of the complex behaviour of cancer stem cells.

This paper focuses on the creation of new types of cerium oxide nanoparticles heavily doped with gadolinium (Ce_0.8_Gd_0.2_O_2−x_) as MRI contrast agents, with the emphasis on their cytotoxicity and biocompatibility with mesenchymal stem cells. Deep insights into the molecular mechanisms of the cytotoxicity of the nanoparticles confirmed the great potential of Gd-doped ceria in MRI imaging, due to its low toxicity to human mesenchymal stem cells. Evidence of the contrast properties of synthesised NPs has emerged from water proton relaxation studies in the presence of Gd-doped ceria, using a high resolution ^1^H 700 MHz nuclear magnetic resonance (NMR) spectrometer equipped with a field cycling apparatus in a magnetic field range from 4 mT to 16.4 T. The Ce_0.8_Gd_0.2_O_2−x_ NPs demonstrated excellent MRI contrast properties.

## 2. Results and Discussion

Using microwave-hydrothermal (Sample 1) and polyol-mediated syntheses (Samples 2, 3), transparent light-yellow aqueous sols were prepared, and showed no visible opalescence, while demonstrating a pronounced Tyndall effect. The X-ray powder diffraction patterns (Figure S1) indicated that the crystal structure of the dried samples coincided well with the structure of CeO_2_ ($Fm\bar{3}m$, PDF2 34-0394). Wide maxima in the diffraction patterns indicate that the particle size in the synthesised materials was rather small; the estimates made using the Scherrer equation amounted to 5 nm for Sample 1 and 2–3 nm for Samples 2 and 3. A full-profile analysis of the X-ray diffraction patterns showed that the crystal lattice parameter values (*a*) for the samples (0.54204(5) nm for Sample 1, 0.5440(2) nm for Sample 2, and 0.5442(5) nm for Sample 3) differed from the lattice parameter of the bulk CeO_2_ (0.54113 nm [29]). According to recently published data, this might have been due to the small (<5 nm) size of ceria nanoparticles [30,31], or the formation of cerium-gadolinium oxide solid solutions (for the same coordination number VIII, Gd^3+^ ionic radius is 0.1053 nm, Ce^4+^ ionic radius is 0.097 nm [32]). The authors’ previously reported data [33,34] proved the formation of CeO_2_:Gd solid solutions under certain synthetic conditions. Energy dispersive X-ray spectroscopy indicated that, within the measurement accuracy, the chemical composition of all samples corresponded well to the chemical formula Ce_0.8_Gd_0.2_O_2−x_, with the Ce:Gd atomic ratio being (79 ± 2):(21 ± 2).

**Figure 1:**
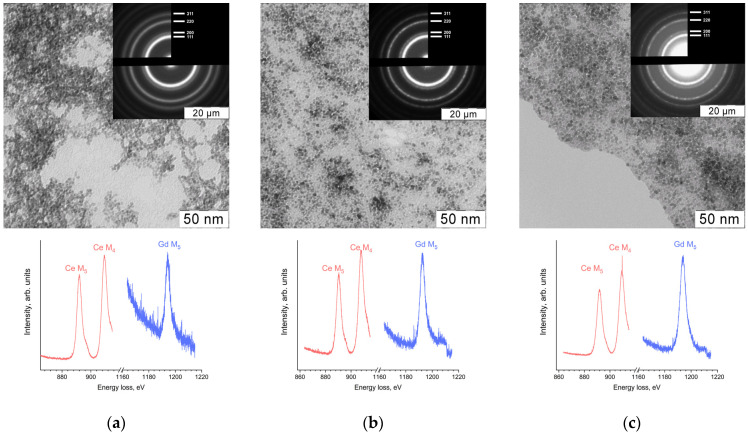
TEM and SAED (top), EELS (bottom) data for the gadolinium-doped ceria aqueous sols: (**a**) Sample 1; (**b**) Sample 2; (**c**) Sample 3.

The results of transmission electron microscopy (TEM), selected area electron diffraction (SAED) and electron energy loss spectroscopy (EELS) showed the formation of nanocrystalline ceria particles with average diameters 4 ± 1 nm for Sample 1 or 3 ± 1 nm for Samples 2 and 3 (Figure 1). EELS data also demonstrated the presence of gadolinium in the samples. In the electron energy loss spectra (Figure 1), the ratio of Ce M_4_ to Ce M_5_ edge peak intensity was approximately 1.2. This fact, along with the positions of the peaks and the presence of the shoulders, indicates the presence of only tetravalent cerium in the materials [35].

**Figure 2:**
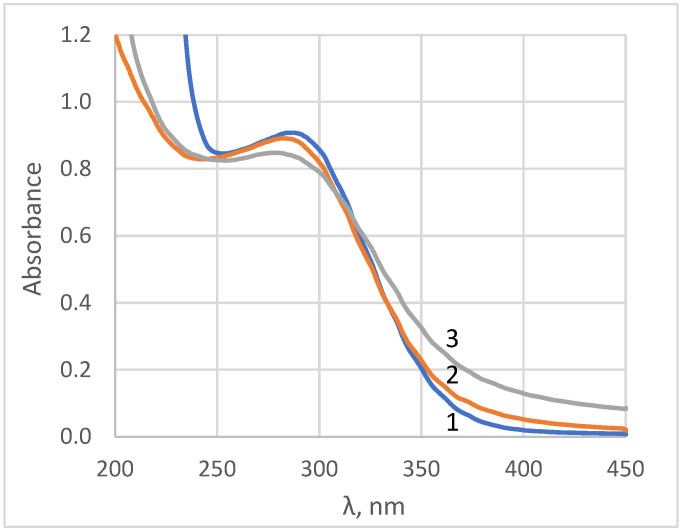
UV-vis spectra of gadolinium-doped ceria sols, Samples 1–3.

The formation of nanocrystalline ceria was also confirmed by UV-vis spectroscopy (see Figure 2). The absorbance spectra are typical of aqueous sols of ceria nanoparticles which have an optical band gap value of 3.0–3.1 eV [36,37]. In the UV-region, the shape of ceria sols’ absorbance spectra was very sensitive to the presence of cerous ions, which had an absorption band at 253.6 nm with a molar extinction coefficient of 685 M^−1^·cm^−1^ [38]. In turn, the absorbance maximum of ceric ions was located at ca. 320 nm, with a molar extinction coefficient of 5580 M^−1^·cm^−1^ [39]. The differentiation of the absorbance spectrum enables the estimation of cerium valence state in aqueous sols [40]. The profile analysis of the absorbance spectra [40] confirmed the absence of trivalent cerium species in the sols.

The Gd-doped ceria sols prepared using the selected protocols exhibited high stability in different media, including Dulbecco’s Modified Eagle Medium/Nutrient Mixture F-12 (DMEM/F12) or phosphate buffer solution (PBS) (see Table 1). When mixed with PBS and/or DMEM/F12 solutions, none of the sols showed any visible opalescence or sedimentation for at least 3 days. A slight increase in the hydrodynamic size of the NPs after incubation with DMEM/F12 containing fetal bovine serum (FBS) can be due to the opsonization of the NPs (the absorption of serum proteins on their surface). The small hydrodynamic size of the NPs in DMEM/F12 (<10 nm) suggests the high stability of the Gd-doped ceria NPs in biological media. Such an ultra-small size makes it possible to ensure high efficiency of endocytosis into various cell types, including hMScs, and can also minimize the risk of an immune response of the body upon their direct injection into the bloodstream. In PBS, the aggregation of the NPs is more pronounced, and is connected to the strong chemisorption of phosphates on the surface of Gd-doped ceria, which affects the bioactivity of NPs [41,42].

Thus, the synthetic protocols enabled the formation of nanocrystalline Gd-doped ceria sols containing 20 at.% Gd, with high colloid stability in media commonly used for in vitro experiments. The main problem in the synthesis of rare earth doped ceria colloids arises from very high differences in the solubility of ceria and rare earth hydroxides. In particular, the solubility of gadolinium hydroxide is more than ten orders of magnitude higher than that of ceria (p*K*_sp_(Gd(OH)_3_) = 27 [43], p*K*_sp_(Ce(OH)_4_) = 42 [44], p*K*_sp_(CeO_2_) = 59 [12]). In this regard, even the sequence of the addition of chemicals into the reaction mixture is meaningful. The addition of an alkali solution to a solution of rare earth salts favours the formation of core-shell structures. Due to the extremely low solubility of ceric hydroxides, under aerobic conditions cerous ions will be oxidised in the first instance, forming a ceria core, and then gadolinium hydroxides will sediment on its surface. Regarding medical applications, such core-shell structures are disadvantageous, due to the presence of relatively soluble gadolinium compounds on the surface of nanoparticles. They can impart toxicity to ceria-based NPs, thus nullifying the main idea of the use of Gd-doped ceria as a non-toxic MRI agent. To achieve greater homogeneity among the NPs and to avoid the formation of core-shell structures, inverse precipitation must be used.

The incorporation of Gd^3+^ ions into the CeO_2_ crystal structure and the formation of crystalline Gd-doped ceria are crucial for their use as non-toxic MRI agents, again, due to the relatively high solubility of gadolinium hydroxides. The formation of the solid solutions requires high temperature processing, e.g., hydrothermal or solvothermal treatment. It should be noted that the high chemical homogeneity of nanoparticles achieved at the precipitation stage will facilitate the crystallisation of CeO_2_:Gd NPs. The use of capping agents (e.g., triethyleneglycol) with good biocompatibility [45] and chelating ability [46] can additionally promote the MRI-contrast properties of gadolinium compounds: an enhancement in the in vitro and ex vivo relaxivity of gadolinium oxide NPs has been shown for glycols with increasing chain length [47].

**Figure 3:**
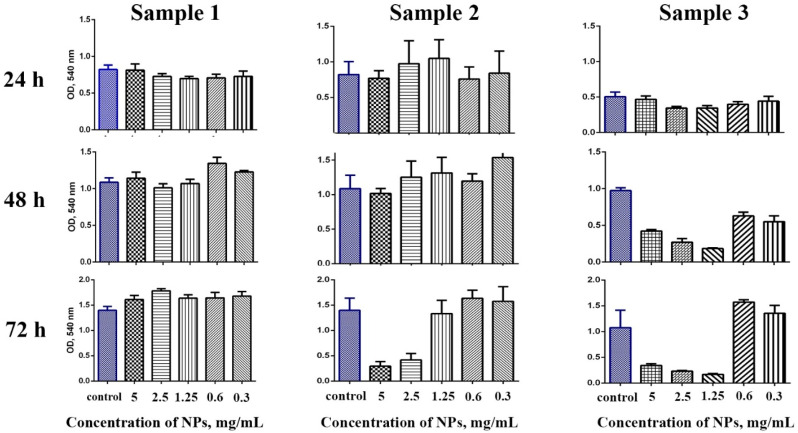
Analysis of hMSc viability according to MTT test. The cells were incubated with Ce_0.8_Gd_0.2_O_2−x_ nanoparticles (0.3–5 mg/mL) for 24, 48 and 72 h.

The cytotoxicity of Gd-doped ceria NPs was assessed by analysing the dehydrogenase activity of human mesenchymal stem cells. The analysis was conducted using 3-[4,5-dimethylthiazol-2-yl]-2,5 diphenyl tetrazolium bromide (MTT) assay upon 24, 48 and 72 h of cell cultivation (Figure 3). For the experiments, the cells were cultivated with extremely high concentrations of the NPs (in the range of 0.3–5 mg/mL), to reliably detect any possible toxic effects. The data indicated that the samples showed different cytotoxicity. Sample 1 did not show any cytotoxic effects throughout the range of concentrations (0.3–5 mg/mL), and these results corroborate the previously reported negligible cytotoxicity of citrate-stabilised ceria NPs [44]. Conversely, Sample 2 and Sample 3 in high concentrations showed a decrease in the cells’ viability after prolonged (48 and 72 h) cultivation. Sample 2 did not show any cytotoxicity after 48 h of cultivation, while for the longer cultivation (72 h) there was a decrease in the cells’ viability at high concentrations of nanoparticles (2.5 and 5 mg/mL). Sample 3 appeared to be the most cytotoxic, its negative effect on stem cells being observed after 24 h of cultivation; when the concentration of NPs decreased to 0.6 mg/mL, however, the sample did not show a pronounced cytotoxic effect, even after 72 h of cultivation with the stem cells.

The increased cytotoxicity of Samples 2 and 3 towards stem cells was most probably connected to the adsorption of the products of triethylene glycol oxidative thermolysis on the cerium oxide NPs’ surface. Despite the low toxicity of triethylene glycol itself [45], heating triethyleneglycol in air can produce various potentially toxic carbonyls and aldehydes [48]. Note that the higher toxicity of Sample 3 correlates well with the longer heating duration during its synthesis.

**Figure 4:**
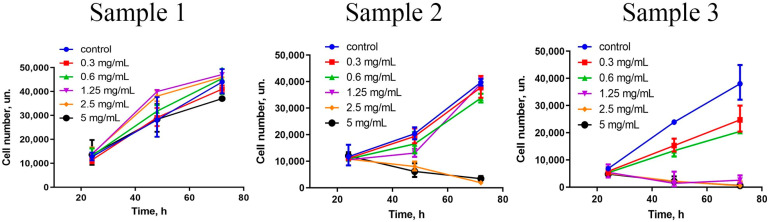
Growth curves of mesenchymal stem cells in the presence of various concentrations (0.3–5 mg/mL) of Ce_0.8_Gd_0.2_O_2−x_ nanoparticles during 24, 48 and 72 h of incubation.

A comprehensive assessment of nanoparticles’ cytotoxicity should include the analysis of the proliferative activity of the cells. For different cells, ceria NPs modified with different surface ligands can either inhibit [49] or stimulate [13] cell migration and proliferation. The data showed (Figure 4) that Sample 3, in the concentration range of 1.25–5 mg/mL, completely inhibited the proliferative activity of hMSc. Sample 2 also inhibited stem cells’ proliferation, but only at high concentrations (2.5 and 5 mg/mL) and after prolonged (72 h) cultivation. At lower concentrations (0.3–1.25 mg/mL), no toxic effect of the NPs was observed. Interestingly, Sample 1 triggered proliferative activity of hMSc after 48 and 72 h of cultivation. The latter observation is in line with previously reported results on the stimulating action of citrate-stabilised ceria NPs [50].

The stimulation of the proliferative activity of hMSc in the presence of citrate-stabilised ceria NPs (Sample 1) was most probably due to their high redox potential, which modulated the cells’ redox status. Oxidative stress is the main reason for the suppression of cell proliferation in vitro [51], since it inevitably arises in cells in response to various forms of manipulation, including isolation and trypsinisation [52]. These negative effects can be levelled by ceria NPs acting as a reactive oxygen species (ROS) scavenger and inactivating the excess of intracellular ROS.

**Figure 5:**
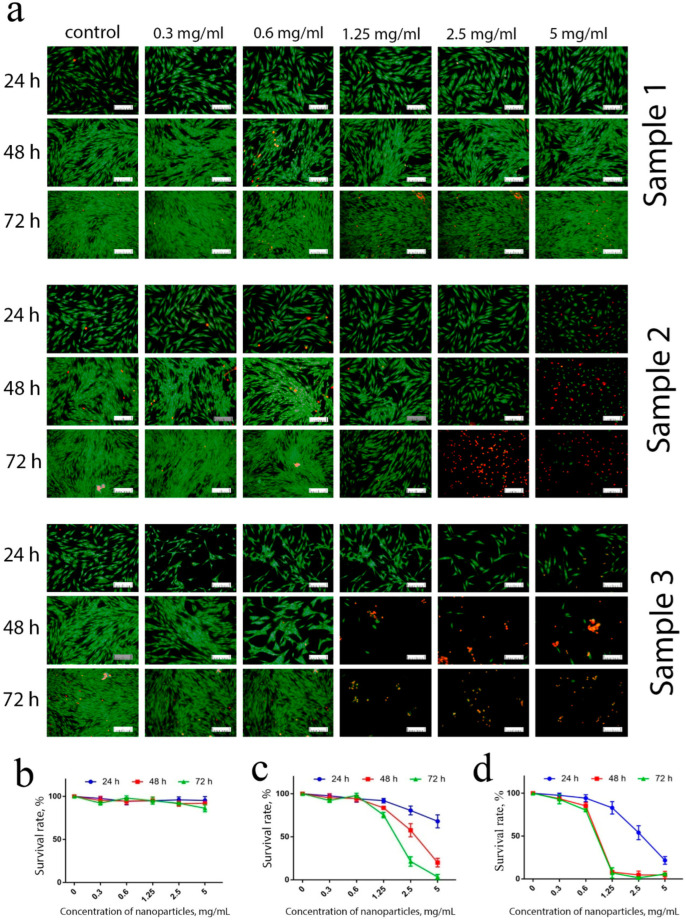
Live/dead assay of hMSc after incubation with Ce_0.8_Gd_0.2_O_2−x_ nanoparticles. Micrographs (**a**) and quantification (**b**–**d**) of viable/dead hMSc after 24, 48 and 72 h of cultivation with Ce_0.8_Gd_0.2_O_2−x_ nanoparticles (0.3–5 mg/mL). All the images were taken at the same magnification, the scale bar is 100 μm.

These results were supported by differential cell staining using live/dead assay (Figure 5). The introduction of low concentrations (0.3–1.25 mg/mL) of Sample 1, as expected, did not result in an increase in the number of dead cells after 24, 48 or 72 h of cultivation. At maximum concentrations (2.5 and 5 mg/mL), an insignificant percentage of dead cells was observed after 72 h of cultivation only.

Conversely, more than 40% of the cells died after 24 h of contact with Sample 2 at maximum concentrations. Sample 3 was the most toxic, since, even at the lowest concentration used (0.3 mg/mL), a reliable increase in the number of dead cells was observed, due to the inhibition of proliferative activity. The observed morphological changes supported the high percentage of apoptotic cells in the culture.

Thus, the ceria nanoparticles coated with triethyleneglycol (TEG) prepared using the solvothermal method (Sample 2 and Sample 3) inhibited the proliferative and dehydrogenase activity of human mesenchymal stem cells (Figure 4 and Figure 5). Further experiments showed that the negative effect of this type of ceria NPs was most probably connected to the changes in redox status in the cells.

**Figure 6:**
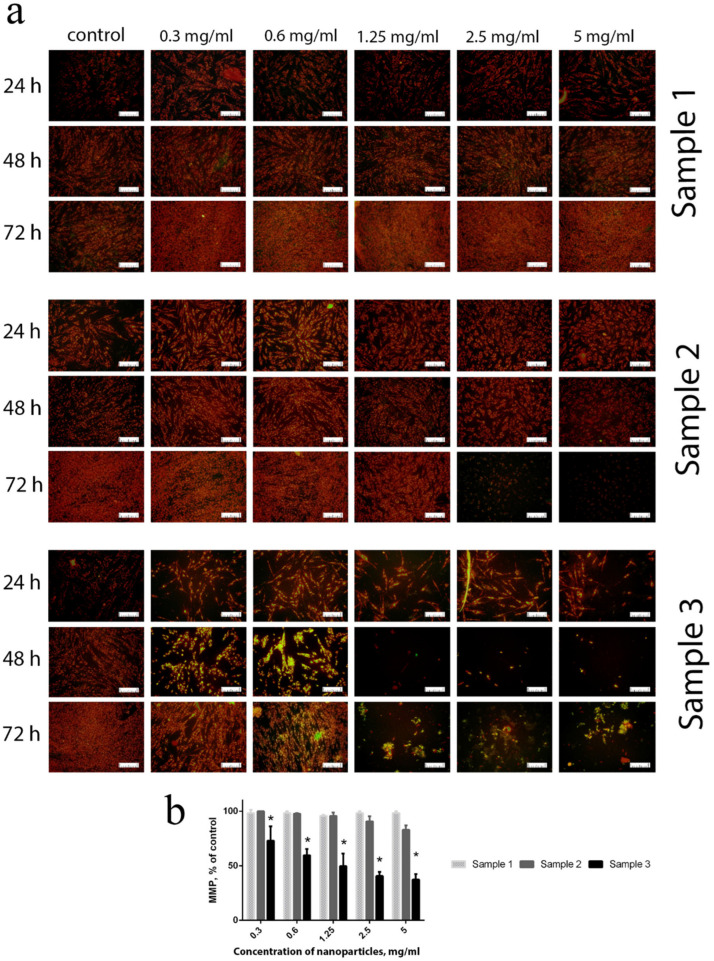
Micrographs (**a**) and quantitative assessment (**b**) of the mitochondrial membrane potential for hMSc after 24, 48 and 72 h of incubation with Ce_0.8_Gd_0.2_O_2−x_ nanoparticles (0.3–5 mg/mL) (staining with JC-1 dye). * *p* < 0.05. Scale bar is 100 µm.

Functional centres in mitochondria are capable of oxygen reduction to superoxide anion-radicals, which is regarded as a source for various active oxygen species. ROS generated by mitochondria are among the key factors that increase intracellular oxidative stress [53]. Intracellular ROS overproduction imbalances the mitochondrial membrane potential (MMP) and results in a decrease in the metabolic activity of the cells [54]. MMP was used as a reliable marker of the redox status of the cells and their metabolic activity. The experiments showed that contact of cells with Sample 3, throughout the whole concentration range (0.3–5 mg/mL), resulted in a dramatic decrease in their MMP after 24 h of cultivation (Figure 6). After 48 h of cultivation, even low concentrations (0.3–0.6 mg/mL) of Gd-doped ceria NPs (Sample 3) resulted in a reliable decrease in MMP. Sample 2 showed minor changes in MMP when high concentrations (2.5–5 mg/mL) of NPs were in contact with the cell culture for a long time (72 h). For Sample 1, no negative effects were observed throughout the whole range of concentrations studied (0.3–5 mg/mL) and for all cultivation times. The latter observation indicates that citrate-stabilised Gd-doped ceria NPs possess very high biocompatibility, and do not change the mitochondrial membrane potential level.

The changes in metabolic activity of mitochondria can result in cell apoptosis, due to ROS overproduction [55]. For instance, in mitochondria, the overproduction of superoxide anion-radical increases the concentration of hydrogen peroxide. In turn, a high H_2_O_2_ content favours lipid peroxidation, decreases the content of antioxidants with low molecular weight and triggers redox-sensitive caspase cascades and cell apoptosis. Because they are redox active, ceria nanoparticles can either induce [56], or inhibit, intracellular [57] oxidative stress. For stem cells, redox status plays a key role in regulating not only their proliferative activity, but also their differentiation or the preservation of their pluripotent status [58].

**Figure 7:**
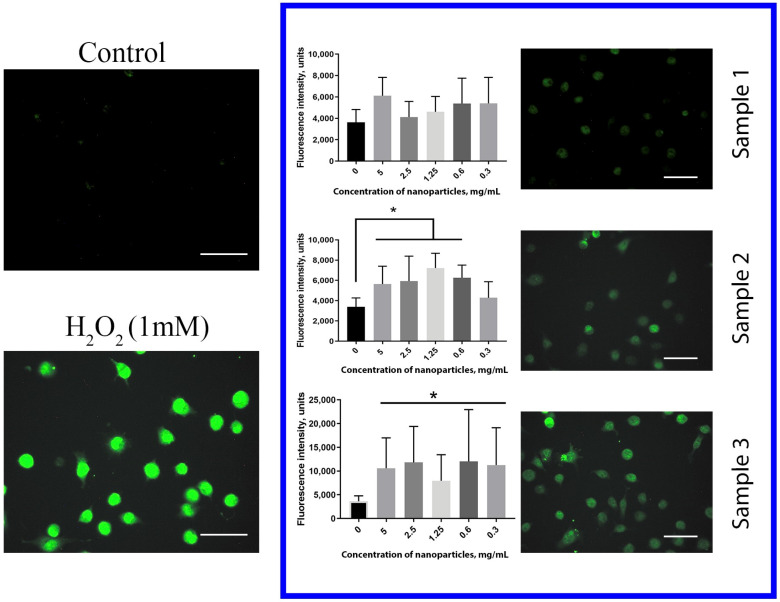
ROS-level assessment after 24 h of incubation with Ce_0.8_Gd_0.2_O_2−x_ nanoparticles; hMSc were labelled with DCF (40 µM) and incubated for 30 min. Cells were analysed using a fluorescent plate reader (Tecan Infinity 200). Mean +/− standard deviation (SD) is plotted for five replicates. * *p* < 0.05. Scale bar is 100 µm.

The level of intracellular ROS in human hMSc was estimated after cultivation with Gd-doped ceria nanoparticles using dichlorofluorescein (DCF) as a fluorescent ROS indicator (Figure 7). The treatment of the cells with 1 mM H_2_O_2_ for 30 min was used as a positive control. The data obtained showed unambiguously that both Sample 2 and Sample 3 dramatically increased the level of intracellular ROS after 24 h of incubation. Cell cultivation with Sample 3, throughout the entire range of concentrations (0.3–5 mg/mL), resulted in a dramatic (more than 3 times) increase in the ROS level. For Sample 2, the increase in ROS concentration was less pronounced than with Sample 3. In turn, no reliable effect of citrate-stabilised Gd-doped ceria NPs (Sample 1) on the intracellular ROS level was observed. The data obtained corroborated the mitochondrial membrane potential measurements, and supported the high biocompatibility of citrate-stabilised Gd-doped ceria NPs.

The cytotoxic effects of nanoparticles resulting in cell apoptosis and death not only arise due to the upregulation of ROS level, but also can involve different mechanisms associated with genotoxic effects, i.e., damage to deoxyribonucleic acids (DNA), ribonucleic acid (RNA) or chromosomes [59,60,61]. For stem cells, genomic instabilities or epigenetic alterations inhibit their further differentiation, and can result in crucial changes to their pluripotent status. Their high sensitivity to any damage, and rapid activation of apoptotic programmes, make them a highly sensitive test-system for the evaluation of the toxicity of nanomaterials [62].

**Figure 8:**
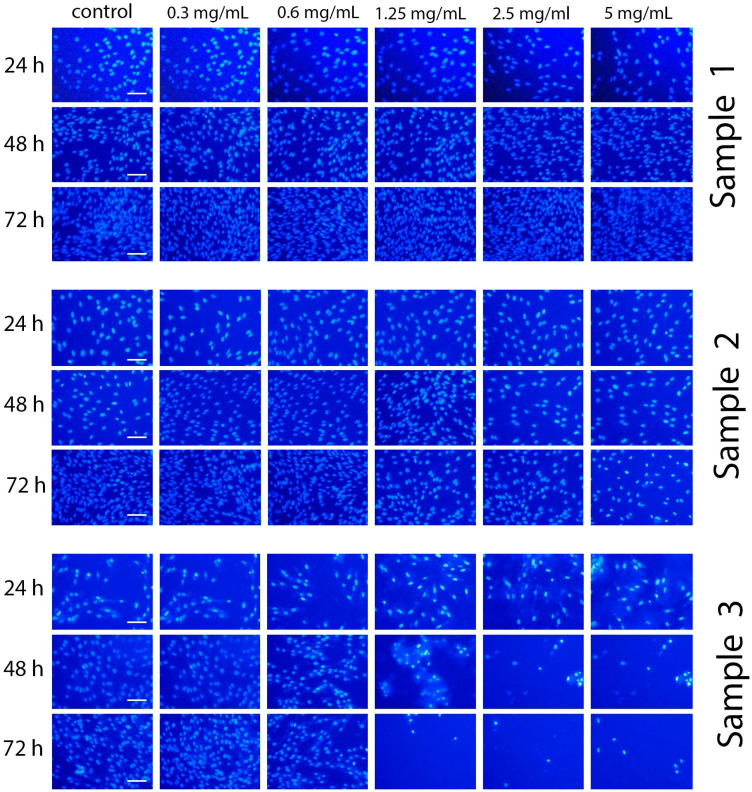
Micrographs of hMSc after 24, 48 and 72 h of incubation with Ce_0.8_Gd_0.2_O_2−x_ nanoparticles (0.3–5 mg/mL) and a quantitative estimation of the proportion of cells with nuclear apparatus abnormalities (fragmentation or change in the morphology of the nucleus) after 72 h of cultivation (staining with Hoechst 33342). All the images were taken at the same magnification. Scale bar is 100 µm.

To assess the genotoxicity of Gd-doped ceria NPs, the morphological status of nuclei was analysed by staining the cells with Hoechst 33342 dye. Images of the stained cells incubated with the NPs are presented in Figure 8. The results show that Sample 3 showed the highest genotoxicity. After 48 and 72 h of incubation with the NPs (1.25, 2.5 and 5 mg/mL), significant changes in chromatin morphology were observed. Even after 24 h of incubation, condensation of chromatin was observed, and some cells contained defragmented chromatin. Conversely, the incubation of the cells with Sample 1 or Sample 2 resulted in neither chromatin condensation nor any anomalies in nuclei morphology. These observations suggest that the latter samples can be attributed to being non-genotoxic.

**Figure 9:**
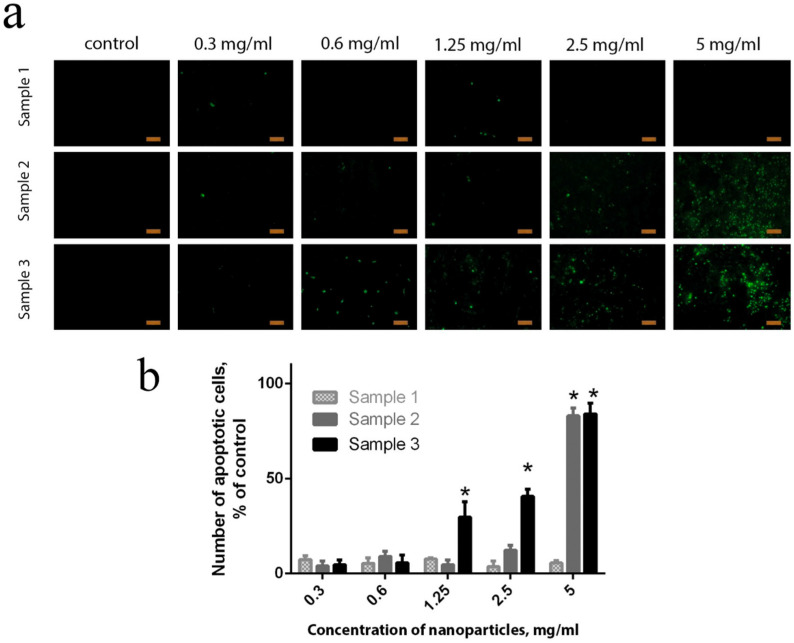
Identification of apoptotic cells after 24 h of incubation with different concentrations of Ce_0.8_Gd_0.2_O_2−x_ NPs. hMSc cells were stained with YO-PRO-1 dye (1 µM) and were further observed under a fluorescence microscope (**a**). A quantitative assessment of apoptotic cells was performed by analysing at least three areas from three different images (**b**). In the control sample, no Ce_0.8_Gd_0.2_O_2−x_ nanoparticles were added. Mean +/− standard deviation (SD) is plotted for five replicates. * *p* < 0.05. Scale bar is 100 µm.

All the cytotoxic effects of the nanoparticles, associated both with ROS regulation and with genotoxicity, result in cell apoptosis and death [63,64]. The number of apoptotic cells in the culture can easily be analysed through staining with Oxazole Yellow iodide (YO-PRO-1) dye. This analysis has shown the result of the integral action of the different mechanisms of the negative action of nanoparticles in living systems. As follows from the data presented in Figure 9, a dose-dependent increase in the number of apoptotic bodies was observed after 24 h of incubation of the human stem cells with TEG-stabilised Ce_0.8_Gd_0.2_O_2−x_ NPs (Sample 2 and Sample 3). A reliable cell apoptosis was observed upon the introduction of Sample 2 at high concentrations (2.5 and 5 mg/mL), and upon the introduction of Sample 3, even at low concentrations (0.6 mg/mL and higher). At the highest concentration (5 mg/mL) of Sample 3, the content of apoptotic cells reached 80%, which corroborated live/dead-assay data (Figure 7) and the results of intercellular ROS concentration measurements (Figure 6). Only a few apoptotic bodies were observed in the cell culture after cultivation with citrate-stabilised Gd-doped ceria NPs (Sample 1), even at the highest concentration (5 mg/mL).

The transcription profile is a very important metabolic indicator of the functional state of hMScs, and is capable of assessing their metabolic state and their ability to actively proliferate and differentiate [65]. Therefore, a detailed analysis was conducted of the level of expression of key hMSc genes involved in the processes of proliferation and differentiation, their redox status, etc. In addition, an analysis of the expression of key pro- and anti-apoptotic genes made it possible to identify some molecular mechanisms of the cytotoxic effect of the Gd-doped ceria NPs.

**Figure 10:**
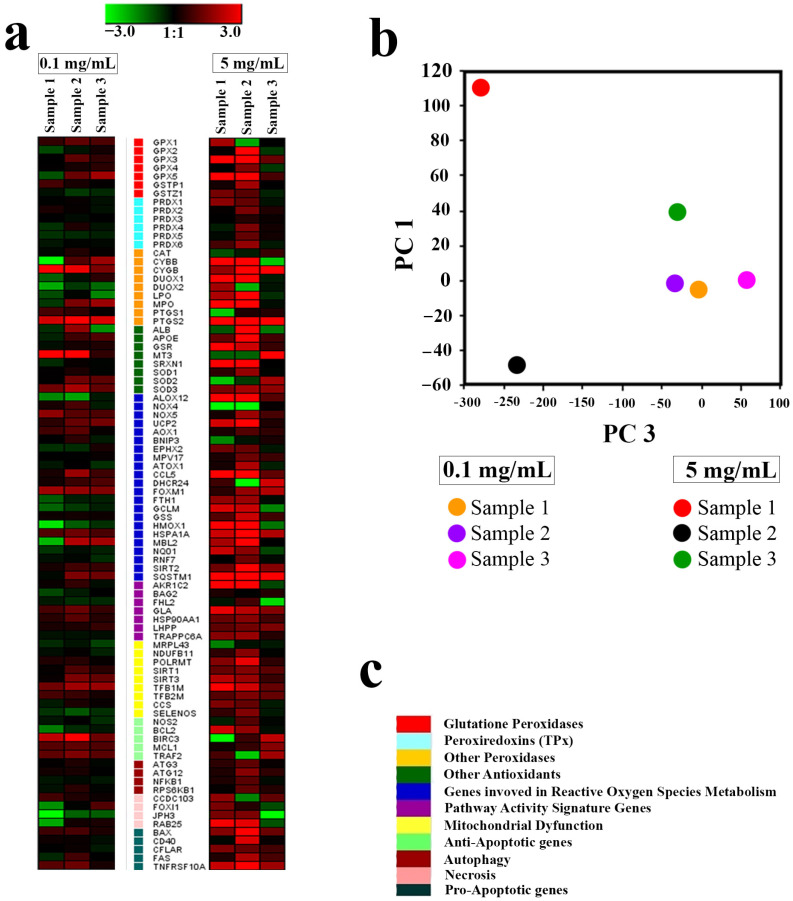
Heat maps of gene expression in hMScs treated with Ce_0.8_Gd_0.2_O_2−x_ NPs (0.1 and 5 mg/mL) after 24 h of incubation (**a**). The intensity scale of the standardised expression values ranges from 3 (green: low expression) to +3 (red: high expression), with a 1:1 intensity value (black) representing the control (non-treated). A principal component analysis (PCA) of RT-PCR data for the cells treated with samples of Ce_0.8_Gd_0.2_O_2−x_ NPs (**b**). Cluster groups of genes and their functionality (**c**).

Further analysis showed that the molecular mechanisms of the cytotoxicity of Gd-doped ceria nanoparticles primarily involved the activation of ROS-signalling pathways for Samples 1 and 2 at high concentrations. Sample 3, which showed the highest toxicity at high concentrations, suppressed the expression of almost all genes. Evidence of this was provided from real-time polymerase chain reaction (RT-PCR) analysis conducted for the panel of 96 genes responsible for cell redox status, antioxidant activity, mitochondrial metabolism, autophagy and apoptosis. All tested samples of NPs showed a dose-dependent response in their expression of the selected genes (see Figure 10). At the lowest concentration (0.1 mg/mL), Sample 1 caused an overexpression of antioxidant genes MT3, PTGS2 and CYGB, and a downregulation of necrosis genes JPH3, FOXI1 and RAB25. The introduction of Sample 2 (0.1 mg/mL) caused a similar overexpression of genes PTGS2, CYGB, MT3 and BIRC3, while the introduction of Sample 3 did not result in changes in expression pattern for the genes MT3 and BIRC3. At high concentrations (5 mg/mL), Sample 1 and Sample 2 caused expression of the entire panel of genes. In turn, Sample 3 (5 mg/mL) did not cause any changes in the expression of the peroxyredoxine (PRDX1-PRDX6), gluthathione peroxidase (GPX1) or gluthathione transferase (GSTP1) genes, nor in the genes associated with the Nf-kB signalling pathway. For Sample 3, a notable downregulation was observed only with a high concentration of NPs, and for a limited number of genes (10). Principal component analysis (PCA) showed that all the tested samples, at a low concentration (0.1 mg/mL), had little effect on the expression pattern of the selected genes (all experimental points are adjacent when orthogonally projected onto a plane), while a high concentration of NPs (5 mg/mL) led to a sharp scattering of experimental points upon orthogonal projection onto a plane (Figure 10b).

**Figure 11:**
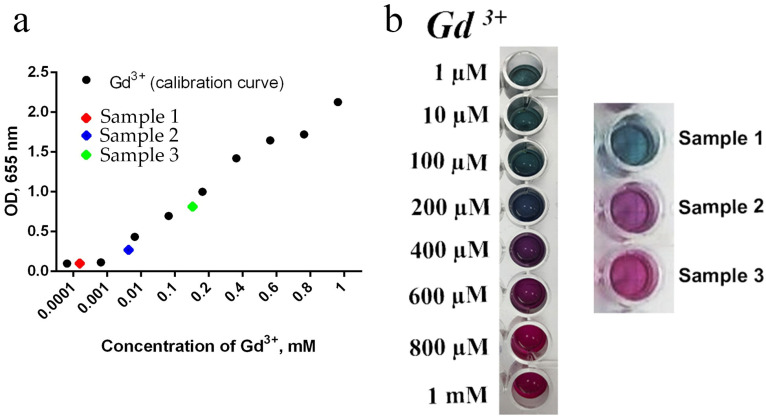
Analysis of free gadolinium content in saline after incubation with Ce_0.8_Gd_0.2_O_2−x_ nanoparticles. (**a**) Optical density (OD) values at 655 nm of the controls (black dots) and of the colloid solution of Ce_0.9_Gd_0.1_O_1.95_ NPs mixed with arsenazo III (red, blue and green dots). (**b**) The appearance of gadolinium(III) nitrate aqueous solutions of various concentrations (0.001–1.000 mM) mixed with arsenazo III, used as controls for the construction of the calibration curve.

A possible reason for the high cytotoxicity of TEG-stabilised Gd-doped ceria nanoparticles is the release of Gd^3+^ ions into the intercellular environment. Generally, the release of highly toxic Gd^3+^ ions is a serious problem in the design of new Gd-containing MRI contrast agents [66,67,68,69]. Gd-doped ceria NPs are generally considered to be extremely low soluble materials which do not release any free Gd^3+^ ions into the environment [8,9,15,16,17,18,19,70]; nevertheless, the assessment of free gadolinium release is very much needed for such nanomaterials. Figure 11 shows the results of the analysis of free gadolinium release from NPs using Arsenazo III assay. Sample 3 was shown to be the most soluble; free gadolinium content in the mother saline solution exceeded 0.1 mM, which might explain the high cytotoxicity of Sample 3. The release of free gadolinium from Sample 2 was much lower ([Gd^3+^] < 0.01 mM), while the Gd^3+^ release from Sample 1 was beyond the detection limit.

Enhanced gadolinium leaching from the NPs synthesised using solvothermal synthesis in glycols can be due to the increased stability and solubility of gadolinium complexes with polyglycol media [71].

**Figure 12:**
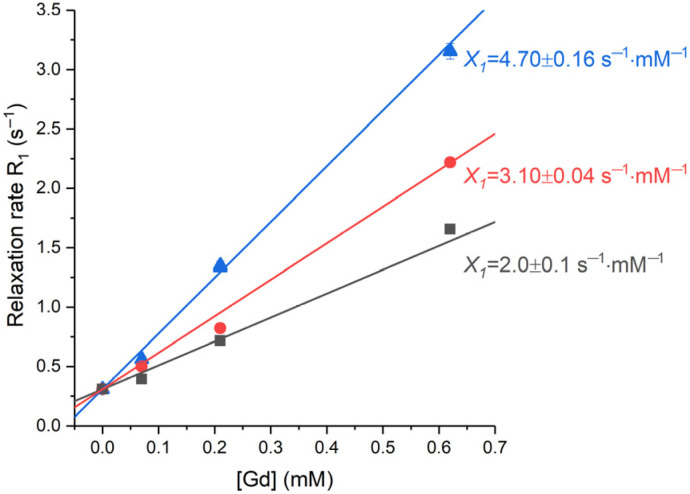
The dependencies of relaxation rate on the concentration of gadolinium for Samples 1–3 at 16.4 T, 27 °C. Black squares—Sample 1, red circles—Sample 2, blue triangles—Sample 3. Lines show the best linear fit of the data; longitudinal-relaxivity values are provided near the corresponding lines.

For the assessment of the magnetic response on the nanoparticles, proton relaxation rates, *R*_1_, were measured for the Samples 1–3, at various concentrations, in a magnetic field of 16.4 T (Figure 12). Standard inversion recovery experiments were conducted for the gadolinium concentrations [Gd] equal to 0.07, 0.21 and 0.62 mM and for the pure water. The resulting set of data was fitted to an exponential growth function (see Section 3.4 for details). These measurements enabled the determination of the relaxivity values (*X*_i_) as the slopes of the linear dependencies of the relaxation rate on gadolinium concentration (Figure 12).

**Figure 13:**
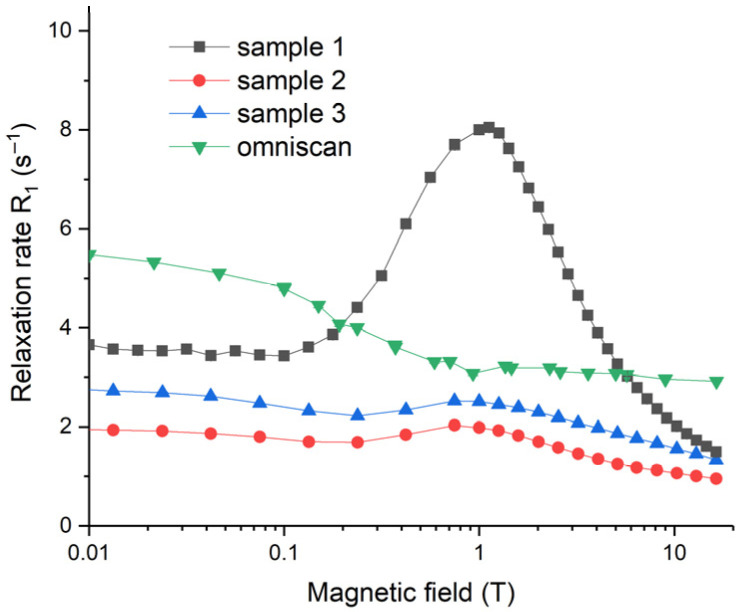
Longitudinal relaxation rates for gadolinium-doped ceria sols (Samples 1–3) and Omniscan solution. The concentration of gadolinium was 0.62 mM for Omniscan and for Sample 1, and 0.21 mM for Samples 2 and 3.

The magnetic field dependencies of water proton longitudinal relaxation rates, *R*_1_, for Samples 1–3, as well as for the gadolinium diamine complex (Omniscan), are presented in Figure 13. Relaxation rates are very sensitive to magnetic field values; they are non-monotonous, and show maxima at particular magnetic field strengths (~1 T), and the maximum is the most pronounced for Sample 1. Such *R*_1_ field dependencies for the aqueous sols of NPs are due to the Curie spin relaxation and for the fluctuations of magnetic moments of NPs around the equilibrium direction [72,73]. A detailed explanation of the observed field dependencies of the relaxation rates requires additional thorough investigation, which is far beyond the scope of this paper. Generally, a nuclear magnetic relaxation dispersion (NMRD) relaxation rate starts to decay when the condition $\omega \tau_{\mathrm{cor}}=1\;$ is met. Here, ω is the nuclear precession frequency and $\;\tau_{\mathrm{cor}}\;$ is a correlation time of water proton motion causing the modulation of magnetic interaction responsible for relaxation. In the case under consideration, *τ*_cor_ reflects the interaction of water protons with either paramagnetic NPs or paramagnetic molecular gadolinium diamine complex (Omniscan). The maxima at ~1 T observed for Samples 1–3 in Figure 13 corresponded to a relaxation process with a correlation time $\tau_{\mathrm{cor}}\approx$ 4 ns. This value is consistent with estimates of the rotational diffusion correlation time for a spherical particle with diameter 4–6 nm in water. For much smaller molecules of the gadolinium diamine complex, the condition $\omega \tau_{\mathrm{cor},\;\mathrm{rot}}\ll 1$ for rotational diffusion was met throughout the entire magnetic field range studied. However, the slow rate of proton exchange in the first coordination sphere of the complex led to a decrease in relaxivity values at fields higher than ca. 0.05–0.1 T, and thus the estimated correlation time was $\tau_{\mathrm{cor},\;\mathrm{ex}}\;\approx$ 200–400 ps.

**Figure 14:**
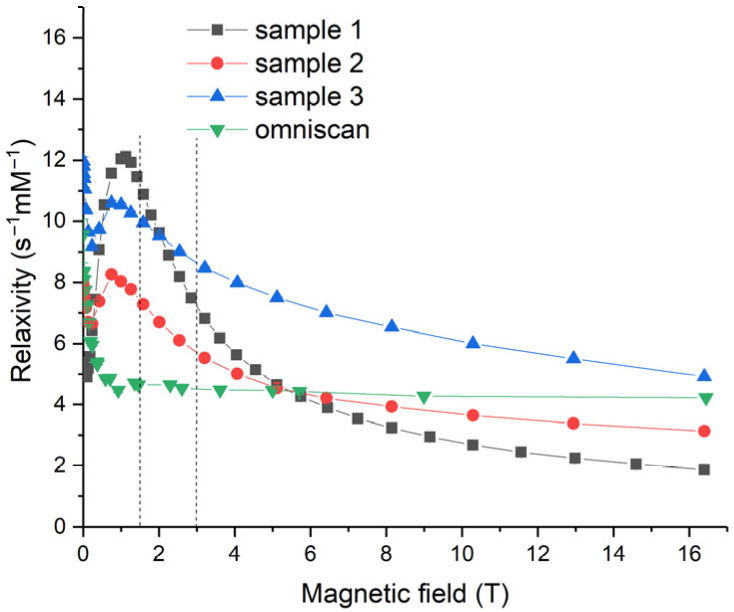
Magnetic field dependencies of relaxivity for the gadolinium-doped ceria nanoparticles (Samples 1–3) and Omniscan. The magnetic field values of 1.4 T and 3.0 T are marked as dashed vertical lines.

Relaxivity values, *X*_1_, offer a more convenient means of tracing the performance of contrast agents. Relaxivity is derived from the relaxation rate, *R*_1_, by bringing it to the concentration of gadolinium atoms [Gd] (see Section 3.4 for details). Relaxivity dependencies on the magnetic field for Samples 1–3 and for Omniscan were calculated using relaxation rate data and Equation (2) in Section 3.4 (see Figure 14). Here, the magnetic fields of 1.4 and 3 T, corresponding to the NMR relaxometer used by Eriksson et al. [19] and the MRI scanner used in the present work, respectively, are marked by vertical dashed lines. It should be noted that the majority of clinical MRI instruments operate at magnetic fields of 1.5 T and 3 T. In a 1.5 T magnetic field, the values of relaxivity were 10.9, 7.3, 9.9 and 4.6 s^−1^·mM^−1^ for Samples 1, 2 and 3, and Omniscan, respectively. In a 3.0 T field, the corresponding values were 7.2, 5.7, 8.6 and 4.5 s^−1^·mM^−1^, respectively. The relaxivity values obtained for the Gd-doped ceria exceeded 10 s^−1^·mM^−1^, showing its excellent performance and suitability for magnetic resonance imaging. A strong relaxation enhancement was observed in the magnetic fields of 0.2 T < *B*_0_ < 6 T for all the Gd_0.2_Ce_0.8_O_2−x_ sols, notably exceeding Omniscan.

The relaxivity of water protons in the presence of Gd-doped ceria nanoparticles strongly depended on the magnetic field. For all the sols, a pronounced relaxivity maximum was observed at ~1 T, followed by a decay at higher magnetic field densities. These data were unavailable in previous reports that discussed MRI materials based on ceria NPs; for example, Eriksson et al. [19] reported relaxivity values at only 1.4 T. Relaxivity field profiles provide both fundamental and practical benefits. Fundamentally, they enable deeper insights into the detailed mechanism of relaxation enhancement for a contrast agent; from the practical point of view, insights into the relaxation enhancement mechanism provide informative feedback for the design of advanced contrast agents.

The most pronounced relaxivity decay with an increase in magnetic field strength was observed for Sample 1, while Omniscan relaxivity was almost constant (4–4.5 s^−1^·mM^−1^) throughout the entire range of field strengths. As the relaxivity decay rate qualitatively correlated with the size of the NPs, these data were in good agreement with the results of the particle size estimations. Sample 1 contained the largest NPs (~5 nm, compared with 2–3 nm for Samples 2 and 3; see above).

**Figure 15:**
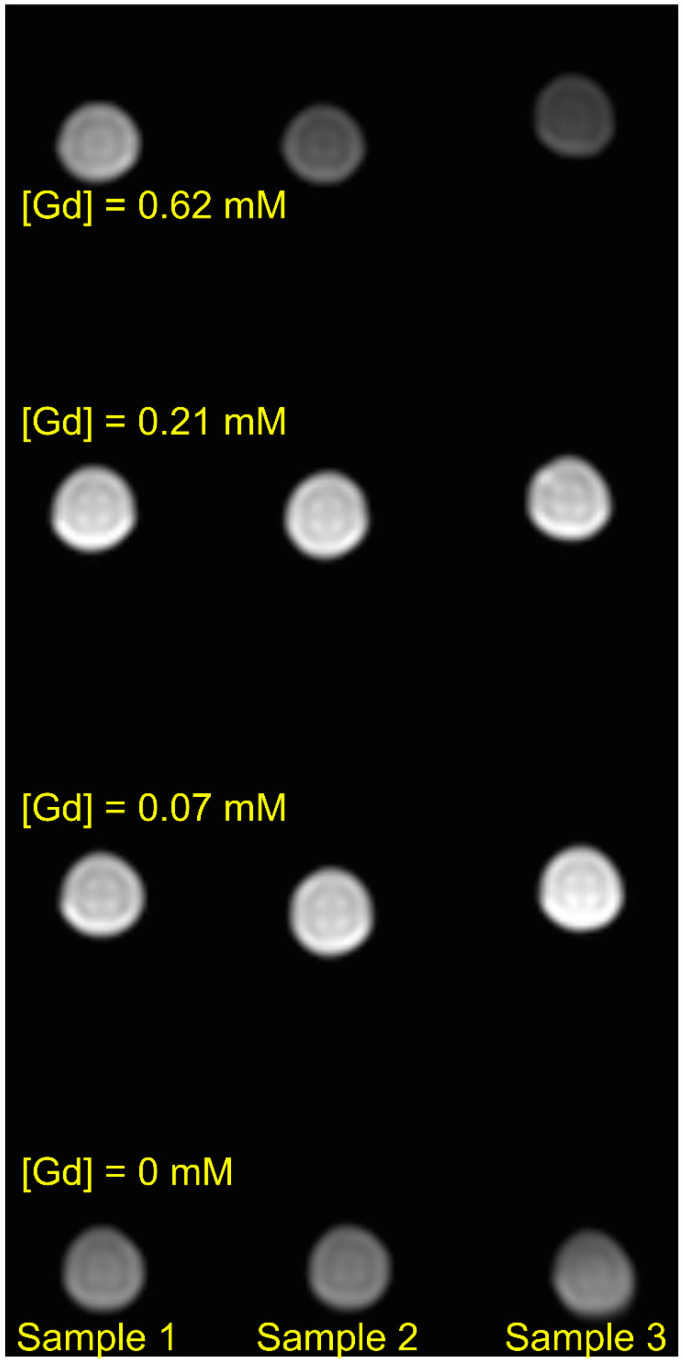
Combined T_1_- and T_2_-weighted image of a cross section of the phantom described in the text. To obtain the image, a T2-TSE pulse sequence was used on a 3 T Philips Ingenia human MRI scanner with parameters TR=2000 ms and TE=200 ms, (the inversion pulse was not applied). Other parameters of the experiment were: 16-channel head coil for the radiofrequency (RF)-pulse application and detection of proton free induction decay, voxel size 0.5 × 0.5 × 10 mm^3^, size of image matrix 160 × 160.

The MRI contrasting ability of Samples 1–3 was demonstrated using T2-Turbo Spin Echo (T2-TSE) pulse sequences [74,75] for the phantom containing nine Eppendorf tubes, with Gd-doped ceria sols with various gadolinium concentrations (0.07, 0.21 or 0.62 mM) and three tubes of pure distilled water. Using various delay parameters, a series of images with combined longitudinal $\;(T_{1})$- and transverse $\;(T_{2})$-relaxation contrast was obtained. An example of such an image is shown in Figure 15. The advantage of MRI images that combine longitudinal- and transverse-relaxation contrast is the high contrast for tissues with different relaxation properties, due to their different viscosity or contrast agent concentrations. Such a high contrast can be achieved with the correct choice of the parameters of the pulse sequence TR (repetition time), TI (delay after 180° radiofrequency inversion pulse), and TE (echo-signal refocusing time). Generally, contrast agents with higher relaxivity show a greater contrast in MRI images. As shown in Figure 15, Sample 1 was the most efficient in terms of relaxivity, providing images with comparable or better contrast throughout the gadolinium concentration range than Samples 2 and 3. In turn, Samples 1–3 provided a better contrast than the pure water (control) samples. These data show that all the Gd-doped ceria NPs are suitable for obtaining bright images in different magnetic relaxation imaging modes. By using Gd-containing sols, image contrast is significantly enhanced and the time necessary for MR image detection is shortened.

## 3. Materials and Methods

### 3.1. Synthesis of Ce_0.8_Gd_0.2_O_2−x_ Nanoparticles

For the synthesis of Gd-doped ceria (Ce_0.8_Gd_0.2_O_2−x_) citrate-stabilised aqueous sols, two previously reported methods were adopted [33,34]. A brief description of the experimental procedures is given below.

Sample 1 was prepared in the following way. Anion-exchange resin Amberlite IRA 410 CL, converted to the OH-form, was gradually added to mixed aqueous solutions of cerium(III) nitrate and gadolinium(III) nitrate (total rare earth concentration was 0.01 M) until pH 10.0 was achieved. As prepared sols were separated from the resin by filtration, they were immediately transferred to polytetrafluoroethylene 100 mL autoclaves (filling degree 50%) and subjected to hydrothermal microwave treatment in a Berghof Speedwave MWS-3+ setup at 150 °C, for 4 h. Temperature was continuously controlled with a built-in IR pyrometer (temperature measurement error ±1 °C). Then, the autoclave was cooled to room temperature in air, and ammonium citrate was added to a prepared sol to give an REE:citrate molar ratio of 1:2.

Samples 2 and 3 were prepared using a polyol method. A total of 50 mL CeCl_3_ and GdCl_3_ mixed aqueous solution was slowly added to an excess of isopropanol/3 M aqueous ammonia (3:1 *v*/*v*). The precipitate was repeatedly washed with distilled water using decantation until a neutral pH was achieved. The obtained suspension was added to triethyleneglycol and boiled under continuous stirring. After the complete removal of water, the mixture was stirred at a boiling temperature of triethyleneglycol (288 °C) for 4 h (Sample 2) or 8 h (Sample 3). Then, 4 wt.% aqueous citric acid was added to the cooled sols mixed with isopropanol (1:1 *v*/*v*), and 5 mL of ammonia (15 M) was added to the mixtures obtained. The precipitate was washed with isopropanol and dried at 60 °C, to remove the excess of the alcohol. Finally, the powders were dispersed in distilled water by ultrasonication.

### 3.2. Methods of Analysis

The size and shape of Ce_0.8_Gd_0.2_O_2−x_ nanoparticles were determined by transmission electron microscopy (TEM) on a Leo912 AB Omega electron microscope equipped with an electron energy loss spectrometer (EELS) at an accelerating voltage of 100 kV.

The chemical composition (energy dispersive X-ray analysis, EDX) of the samples was analysed on a Carl Zeiss NVision 40 field-emission scanning electron microscope equipped with an Oxford Instruments X-MAX (80 mm^2^) detector, at an accelerating voltage of 20 kV. The chemical composition of the samples was also determined, using a simultaneous Thermo Scientific iCAP PRO XP inductively coupled plasma optical emission spectrometer (ICP-OES) with a charge injection device array detector.

The hydrodynamic diameter and ζ-potential of Ce_0.8_Gd_0.2_O_2−x_ NPs was measured using a Malvern Zetasizer Nano ZS Analyser. Before the measurements, the Ce_0.8_Gd_0.2_O_2−x_ sols were diluted (1:10 *v*/*v*) with distilled water.

The leaching of gadolinium ions from NPs into aqueous solution was analysed using absorption spectrometry with arsenazo III assay [76]. Before the analysis, the sol was stirred overnight in 0.9 wt.% NaCl solution and dialysed using a 5 kDa membrane.

The UV-absorption spectra were registered using a Cary 100 UV-visible spectrophotometer, in the wavelength range of 200 to 800 nm.

The measurements were performed using the equipment of the Joint Research Centre for Physical Methods of Research at the Kurnakov Institute of General and Inorganic Chemistry of the Russian Academy of Sciences (JRC PMR IGIC RAS).

### 3.3. Biocompatibility and Biochemical Studies

Human mesenchymal stem cells (hMSc) were isolated from the third-molar germ (pulp), extracted for orthodontic reasons, from a healthy 16-year-old patient. The cells were washed from the dental pulp with DMEM (PanEko, Moscow, Russia) containing 200 U/mL penicillin and 200 μg/mL streptomycin (Life Technologies, Waltham, MA, USA), using a syringe inserted into the dental apex and dissociated by treatment with 0.25% trypsin and 0.02% EDTA (PanEko) for 30 min, at 37 °C. The isolated cells were centrifuged for 3 min at 200× *g* and transferred into culture medium consisting of DMEM/F-12 (1:1; Life Technologies), 10% fetal bovine serum (FBS), 2 mM L-glutamine, 200 U/mL penicillin and 200 μg/mL streptomycin. After attaining subconfluence, the cell cultures were treated with trypsin–EDTA (0.25%) and transferred into new flasks at a ratio of 1:2. In the experiments, three to five passage cell cultures were used. After 6 h, following cell attachment, the medium was substituted with a fresh portion containing Ce_0.8_Gd_0.2_O_2−x_ NPs (0.3–5 mg/mL). In control experiments, the cells were incubated in a culture medium without the addition of Ce_0.8_Gd_0.2_O_2−x_ NPs.

Cell viability was assessed using MTT assay based on the reduction of a colourless tetrazolium salt (3-[4,5-dimethylthiazole-2-yl]-2,5-diphenyl tetrazolium bromide, MTT). Following 48 h culturing, 0.5 mg/mL MTT reagent was added to the wells. The optical density of the formed formazan was measured at *λ* = 540 nm, using a BioRad 680 photometer.

The growth rate of the cell culture after incubation with Ce_0.8_Gd_0.2_O_2−x_ NPs was estimated by counting the number of the cells stained with Hoechst 33342. At least four measurements were made for each Ce_0.8_Gd_0.2_O_2−x_ concentration (0.3–5 mg/mL). The data are presented as growth curves.

The ratio of live and dead cells in the culture was evaluated using a LIVE/DEAD BacLight Bacterial Viability Kit (Invitrogen), containing SYTO 9 dye (stains all cells, *λ* = 485/498 nm) and propidium iodide (stains the nuclei of dead cells, *λ* =535/617 nm). Cell staining was carried out by substituting DMEM/F12 culture medium with 5% FBS with the medium containing a mixture of dyes at a concentration of 1 µg/mL. The morphology of the cells was analysed using an inverted microscope Axiovert 200 (Carl Zeiss) at a 10× magnification. Images were taken with a digital camera, Canon Power Shot A620. The number of viable cells (showing green fluorescence, due to the presence of SYTO 9) and dead cells (showing red fluorescence, due to the presence of propidium iodide) was measured using Image J software (v. 1.53t).

Mitochondrial membrane potential (MMP) was determined by JC-1 dye, using fluorescence microscopy. The JC-1 accumulates in the mitochondrial membrane in a potential-dependent manner. The high potential of the inner mitochondrial membrane facilitates the formation of the dye aggregates (J-aggregates), with both excitation and emission shifted towards red light wavelengths (530 nm/590 nm) when compared with those for JC-1 monomers (485 nm/538 nm). Cells were seeded into 96-well tissue culture plates (Greiner) at a density of 5 × 10^4^ cells per well, and cultured in a CO_2_ incubator at 37 °C for 24, 48 and 72 h with different concentrations of Ce_0.8_Gd_0.2_O_2−x_ NPs. The cells were preincubated with 5 μM JC-1 in the Hanks’ Balanced Salt Solution (HBSS) in a CO_2_ incubator at 37 °C, for 30 min. At the end of the incubation, cells were stained with 10 µM JC-1 in HBSS and incubated again at 37 °C for 30 min. Next, the cells were washed twice, using HBSS and analysed using a Zeiss 200 M inverted fluorescence microscope at 200× magnification. Results were presented as a ratio of fluorescence intensity, i.e., that measured at 530 nm/590 nm (aggregates) to that measured at 485 nm/538 nm (monomers).

Hoechst 33342, a blue fluorescence dye, stains chromatin in normal, apoptotic and necrotic cells. It stains the condensed chromatin in apoptotic cells more brightly than the chromatin in normal cells. The number of apoptotic cells after incubation with Ce_0.8_Gd_0.2_O_2−x_ NPs was measured by direct counting of the number of cells with condensed chromatin on fluorescent microphotographs.

The level of intracellular reactive oxygen species (ROS) was determined using dichlorofluorescein (DCF). Cells were preincubated with Ce_0.8_Gd_0.2_O_2−x_ NPs at various concentrations (0.3–5 mg/mL) for 6, 12 and 24 h in a 96-well plate, and then the culture medium was replaced with Hanks solution containing DCF (10 µM). Untreated cells were used as a negative control. Fluorescence was detected using a Tecan 200 PRO (Tecan Corp., Männedorf, Switzerland) plate reader.

For the RT-PCR experiments, a kit for mRNA isolation with magnetic particles was used according to the manufacturer’s protocol (Sileks, Moscow, Russia). Reverse transcription was carried out using a kit supplied by Sileks (Moscow, Russia) using oligo(dT) primer, according to the manufacturer’s protocol. The derived cDNA was used as a template in RT-PCR. The reaction was conducted using a PCR mixture with SybrGreen (Syntol, Moscow, Russia) in a Biorad CFX-96 thermal cycler or an Applied Biosystems ABI 7500 Fast Real-Time PCR System. The expression level of 96 genes, responsible for 25 key cellular processes, was estimated (Table S1). The analysed genes were selected using the database http://www.sabiosciences.com/ (accessed on 13 June 2022) for PCR profiling of different biological processes. The transcription level was normalised by the expression of housekeeping genes encoding β-actin, RPLP0 (ribosomal protein, large, P0) and GAPDH (glyceraldehyde-3-phosphate dehydrogenase). Gene specific primers were picked using Primer Express software (Applied Biosystems, Waltham, MA, USA). Each measurement was performed in two replications (internal replication) and averaged for two independent samples. Samples without reverse transcription were used as a control. Analysis of the expression data was performed using online services at http://www.sabiosciences.com/ (accessed on 13 June 2022), Mayday v.2.14 software (Center for Bioinformatics Tübingen, Germany) and Genesis software (v.2.28.0) [77].

### 3.4. NMR Relaxometry and Magnetic Relaxation Dispersion Experiments

For the study of the relaxation properties of the Gd-doped ceria sols over a wide range of magnetic fields, a custom engineered fast-field cycling setup was used, based on a high resolution 700 MHz NMR spectrometer (Bruker, Billerica, MA, USA) at the International Tomography Center (Novosibirsk, Russia). This setup was an improvement on the analogous add-on apparatus to the 400 MHz spectrometer that was used in the authors’ previous studies [78]; this enabled a magnetic field variation from 4 mT to 16.4 T. For the study of relaxivity, the dependence of water proton relaxation in external magnetic fields was analysed at various concentrations of the sols. In all NMR experiments, the samples containing Gd-doped ceria sols were placed in standard 5 mm NMR sample tubes.

**Figure 16:**
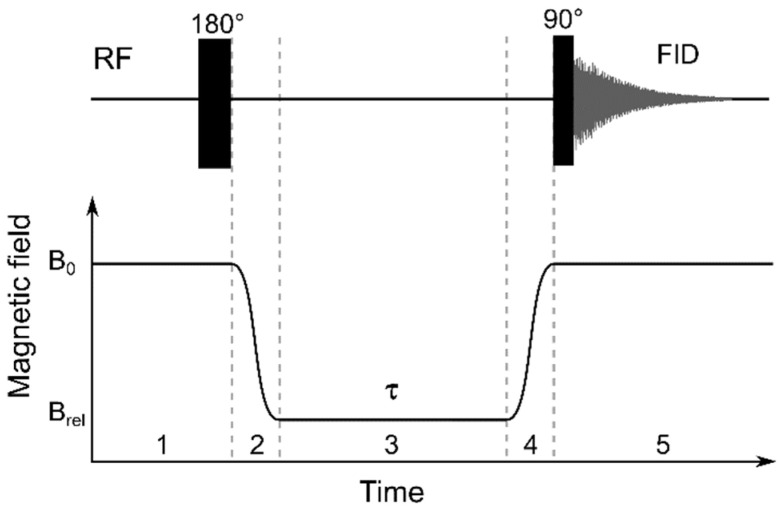
Five-step protocol for nuclear magnetic relaxation dispersion (NMRD) study, combining the high field magnetisation inversion recovery method (*B*_0_ = 16.4 T) (step 1) with fast field cycling in the variable magnetic field *B*_rel_ (4 mT < *B*_rel_ ≤ 16.4 T, step 2), remaining at *B*_rel_ for a variable time interval *τ* (step 3) and returning to *B*_0_ (step 4). The upper line shows radiofrequency (RF) pulses (at steps 1 and 5) for free induction decay (FID) detection synchronised with the mechanical shuttling of the NMR sample along the warm bore of the superconducting magnet of the NMR spectrometer.

For the *T*_1_ measurements in the fixed magnetic field (*B*_0_ = 16.4 T), an inversion recovery pulse sequence (180° − *τ* − 90°) was used with a variable delay, *τ*. For the study of *T*_1_ dependence in a variable magnetic field, *T*_1_(*B*_rel_), a five-step protocol [78] was used, as shown in Figure 16.

In step 1, nuclear spins relaxed at a high field, *B*_0_ = 16.4 T, which is the detection field of a high resolution 700 MHz NMR spectrometer; after that, spin magnetisation was inverted, by applying a 180° pulse. In step 2, the field changed quickly to the relaxation field, *B*_0_ → *B*_rel_, by mechanical positioning of the sample in the fringe field of the superconducting magnet of the NMR spectrometer. In step 3, the spins relaxed at the *B*_rel_ field during a variable time interval, *τ*. For the detection of the resulting spin magnetisation, in step 4, the field changed again, *B*_rel_ → *B*_0_, and in step 5, free induction decay (FID) was measured with a 90° RF pulse. A Fourier transform of FID resulted in an NMR spectrum with an exponential dependence of the signal intensity on *τ* with the characteristic time, *T*_1_. The measured resulting signal intensity as a function of the relaxation delay, τ, was fitted to the following function:

(1) $$I\left(\tau \right)=I_{0}+A\exp\left[-\frac{\tau }{T_{1}}\right].$$

It this way, *T*_1_ values were measured for a set of *B*_rel_ fields to obtain the *T*_1_ magnetic field dependencies, NMRD (nuclear magnetic relaxation dispersions), *T*_1_(*B*_rel_). To compare the relaxation properties of water molecules in the presence of MRI-active species (e.g., nanoparticles), it is more convenient to analyse not the relaxation times (*T*_1_), but the relaxation rates (*R*_1_ = 1/*T*_1_). Linear regression analysis of the relaxation rates at various gadolinium concentrations, [Gd], is a common approach for determining relaxivity per gadolinium atom (*X*_1_). However, to avoid tedious and time consuming acquisition of NMRD data for each sample with various gadolinium concentrations, relaxivity values ($X_{1}$) were calculated at each field, *B*_rel_, from the experimental dependencies, *T*_1_(*B*_rel_), according to the equation:

(2) $$X_{1}=\frac{R_{1}\left(B_{\mathrm{rel}}\right)-R_{1}^{0}}{\left[\mathrm{Gd}\right]}.$$

Here, $R_{1}^{0}$ = 0.294 s^−1^ represents the nuclear relaxation rate of protons in pure water, which was derived from the experimentally measured value $T_{1}^{0}$ = 3.4 s (the relaxation time of protons in pure water); this value does not depend on the magnetic field. Relaxivity values obtained at 16.4 T by linear regression analysis of the relaxation data at different sample concentrations, and by fitting to Equation (2), showed excellent agreement, verifying the correctness of the approach used.

### 3.5. MRI Imaging

For magnetic resonance imaging experiments, a 4 × 3 array of standard 1.5 mL Eppendorf tubes was prepared, and different sols were placed in separate columns. Each tube was filled with 1.5 mL of a sample; gadolinium concentrations decreased from the top to the bottom row of the array. The bottom row of the phantoms contained Eppendorf tubes filled with distilled water. The 2D-MRI images of the phantoms were obtained using a turbo spin-echo (T2-TSE) method on a 3T Philips Ingenia MRI scanner, according to the RLSQ procedure [74].

The *T*_1_, *T*_2_-weighted images and mixed contrast images were obtained by varying the repetition time, TR, between 2000 and 4000 ms, and the echo-signal refocusing time, TE, from 50 to 200 ms, in steps of 50 ms; some of them also used an inversion pulse with a delay after inversion, TI = 1000 ms. A coronal slice (voxel size 0.5 × 0.5 × 10 mm^3^, matrix 160 × 160 and 16-channel head coil) were used in the experimental setup.

## 4. Conclusions

Gadolinium-doped ceria nanoparticles in the form of stable aqueous sols were synthesised using two different techniques, namely anion-exchange hydrolysis of cerium(III) nitrate aqueous solution followed by hydrothermal-microwave treatment, and polyol solvothermal synthesis in triethyleneglycol. The sols possessed excellent stability in different media commonly used for in vitro experiments, including PBS and DMEM. A detailed comparative analysis of the cytotoxicity of these materials was conducted on human mesenchymal stem cells, using different techniques, including viability and growth rate assessment, cell morphology, mitochondrial membrane potential and gene expression analysis, studies of genotoxicity and the level of intracellular reactive oxygen species. The results showed excellent biocompatibility of gadolinium-doped ceria nanoparticles synthesised using anion-exchange and the hydrothermal method. The leaching of gadolinium ions from these nanoparticles was also negligible, supporting their low toxicity. Gadolinium-doped ceria synthesised using the polyol solvothermal technique possessed increased toxicity toward stem cells, which is presumably related to a notable leaching of gadolinium ions from these nanoparticles. All the nanoparticles provided bright MRI images on a 3 T Philips Ingenia scanner, and possessed high relaxivity values, exceeding 10 s^−1^·mM^−1^, thus showing excellent performance and suitability for magnetic resonance imaging. A strong relaxation enhancement was observed in the magnetic fields of 0.2 T < *B*_0_ < 6 T for all the Gd_0.2_Ce_0.8_O_2−x_ sols, notably exceeding Omniscan.

## Figures and Tables

**Table 1:** Hydrodynamic radii of the particles in the gadolinium-doped ceria sols in different media, as measured using dynamic light scattering. The results are presented as mean ± SD.

	MQ Water	PBS Solution (pH 7.2)	DMEM/F12 (pH 7.0–7.3)	DMEM/F12 + 5% FBS (pH 7.0–7.3)
Sample 1	4.5 ± 1.2	19.3 ± 2.8	5.8 ± 2.8	8.5 ± 2.3
Sample 2	3.9 ± 0.4	12.2 ± 2.3	6.0 ± 3.0	5.6 ± 2.6
Sample 3	4.1 ± 2.3	6.9 ± 3.5	8.9 ± 1.2	4.8 ± 1.9

## Data Availability

Data is contained within the article or Supplementary Materials.

## Supplementary Materials

The following supporting information can be downloaded at https://www.mdpi.com/article/10.3390/molecules28031165/s1, Figure S1: XRD patterns of gadolinia-doped ceria; Figure S2: Comparison of the relaxivity field dependencies for Sample 1, measured in two independent series of experiments, using a 400 MHz field cycling setup and a 700 MHz field cycling setup; Table S1: Selected gene groups for RT-PCR analysis.

## Sample Availability

Samples of gadolinium-doped ceria colloid solutions are available from the authors.
