# Supplementary material for: Heavily Gd-Doped Non-Toxic Cerium Oxide Nanoparticles for MRI Labelling of Stem Cells
Source: Molecules. 2023 Jan 24;28(3):1165. doi: 10.3390/molecules28031165 (PMC9920480; doi:10.3390/molecules28031165)
Supplement: Supplementary file 1 [file molecules-28-01165-s001.zip › molecules-2107141-supplementary.pdf]

Supplementary materials

Heavily Gd-doped non-toxic cerium oxide nanoparticles for MRI labelling of stem cells

Anton L. Popov [1], Irina V. Savintseva [1], Taisiya O. Kozlova [2], Olga S. Ivanova [2], Ivan V. Zhukov [3], Alexander E. Baranchikov [2], Alexandra V. Yurkovskaya [3], Andrey A. Savelov [3], Artem M. Ermakov [1], Nelli R. Popova [1], Konstantin L. Ivanov [3] and Vladimir K. Ivanov [1,2*]

[1] Institute of Theoretical and Experimental Biophysics of the Russian Academy of Sciences, 3 Institutskaya st., Pushchino 142290, Russia
[2] Kurnakov Institute of General and Inorganic Chemistry of the Russian Academy of Sciences, 31 Leninskiy prosp., Moscow 119991, Russia
[3] International Tomography Center, Siberian Branch of the Russian Academy of Sciences, 3A Institutskaya st., Novosibirsk 630090, Russia
* Correspondence: van@igic.ras.ru

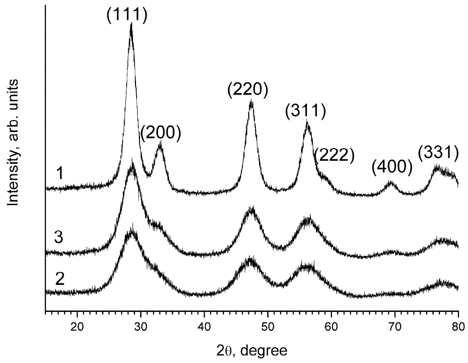

Figure S1. XRD patterns of gadolinia-doped ceria synthesised using different protocols. 1 – Sample 1; 2 – Sample 2; 3 – Sample 3.

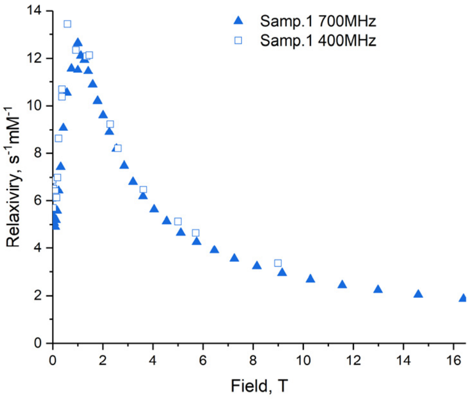

Figure S2. Comparison of the relaxivity field dependencies for Sample 1, measured in two independent series of experiments, the first one using a 400 MHz field cycling setup (open squares), and then, two weeks later, the second one using a 700 MHz field cycling setup (solid triangles). The data are in very good agreement with each other, confirming the stability of the sample and the correctness of the chosen experimental approach.

Table S1. Selected gene groups for RT-PCR analysis.

	Gene #	Gene Symbol
Glutathione Peroxidases (GPx)	A01	GPX1
	A02	GPX2
	A03	GPX3
	A04	GPX4
	A05	GPX5
	A06	GSTP1
	A07	GSTZ1
Peroxiredoxins (TPx)	A08	PRDX1
	A09	PRDX2
	A10	PRDX3
	A11	PRDX4
	A12	PRDX5
	B01	PRDX6
Other Peroxidases	B02	CAT
	B03	CYBB
	B04	CYGB
	B05	DUOX1
	B06	DUOX2
	B07	LPO
	B08	MPO
	B09	PTGS1
	B10	PTGS2
Other Antioxidants	B11	ALB
	B12	APOE
	C01	GSR
	C02	MT3
	C03	SRXN1
	C04	SOD1
	C05	SOD2
	C06	SOD3
Genes Involved in Reactive Oxygen Species (ROS) Metabolism	C07	ALOX12
	C08	NOS2
	C09	NOX4
	C10	NOX5
	C11	UCP2
	C12	AOX1
	D01	BNIP3
	D02	EPHX2
	D03	MPV17
	D04	ATOX1
	D05	CCL5
	D06	DHCR24
	D07	FOXM1
	D08	FTH1
	D09	GCLM
	D10	GSS
	D11	HMOX1
	D12	HSPA1A
	E01	MBL2
	E02	NQ01
	E03	RNF7
	E04	SIRT2
	E05	SQSTM1
Pathway Activity Signature Genes	E06	AKR1C2
	E07	BAG2
	E08	FHL2
	E09	GLA
	E10	HSP90AA1
	E11	LHPP
	E12	TRAPPC6A
Mitochondrial Dysfunction	F01	MRPL43
	F02	NDUFB11
	F03	POLRMT
	F04	SIRT1
	F05	SIRT3
	F07	TFAM
	F08	TFB1M
	F09	TFB2M
	F10	CCS
	F11	SELENOS
Anti Apoptotic	F12	NOS2
	G01	BCL2
	G02	BIRC3
	G03	MCL1
	G04	TRAF2
Autophagy	G05	ATG3
	G06	ATG12
	G07	NFKB1
	G08	RPS6KB1
Necrosis	G09	CCDC103
	G10	FOXI1
	G11	JPH3
	G12	RAB25
Pro apoptotic	H01	BAX
	H02	CD40
	H03	CFLAR
	H04	FAS
	H05	TNFRSF10A
